# Gentamicin at sub-inhibitory concentrations selects for antibiotic resistance in the environment

**DOI:** 10.1038/s43705-022-00101-y

**Published:** 2022-03-30

**Authors:** Concepcion Sanchez-Cid, Alexandre Guironnet, Christoph Keuschnig, Laure Wiest, Emmanuelle Vulliet, Timothy M. Vogel

**Affiliations:** 1 Environmental Microbial Genomics, Laboratoire Ampère, Ecole Centrale de Lyon, Université de Lyon, Ecully, France; 2 Promega France, 69100 Charbonnières-les-Bains, France; 3 Univ Lyon, CNRS, Université Claude Bernard Lyon 1, Institut des Sciences Analytiques, UMR 5280, 5 Rue de la Doua, F-69100 Villeurbanne, France

**Keywords:** Antibiotics, Microbial ecology, Water microbiology, Metagenomics

## Abstract

Antibiotics released into the environment at low (sub-inhibitory) concentrations could select for antibiotic resistance that might disseminate to the human microbiome. In this case, low-level anthropogenic sources of antibiotics would have a significant impact on human health risk. In order to provide data necessary for the evaluation of this risk, we implemented river water microcosms at both sub-inhibitory and inhibitory concentrations of gentamicin as determined previously based on bacterial growth in enriched media. Using metagenomic sequencing and qPCR/RT-qPCR, we assessed the effects of gentamicin on water bacterial communities and their resistome. A change in the composition of total and active communities, as well as a gentamicin resistance gene selection identified via mobile genetic elements, was observed during a two-day exposure. We demonstrated the effects of sub-inhibitory concentrations of gentamicin on bacterial communities and their associated resistome in microcosms (simulating in situ conditions). In addition, we established relationships between antibiotic dose and the magnitude of the community response in the environment. The scope of resistance selection under sub-inhibitory concentrations of antibiotics and the mechanisms underlying this process might provide the basis for understanding resistance dispersion and associated risks in relatively low impacted ecosystems.

## Introduction

Most of the antibiotics used in human therapy and animal production are excreted unaltered to the environment [1]. The selective pressure imposed by environmental pollution with residual concentrations of antibiotics due to anthropogenic activities might result in the development and selection of antibiotic resistance in environmental settings [2, 3] and the subsequent dissemination of antibiotic resistant bacteria (ARB) and antibiotic resistance genes (ARGs) from the environment to animal and human microbiomes [4, 5]. Nonetheless, the scope of this phenomenon remains unclear [6–8].

Antibiotic concentrations found in anthropogenically-polluted environments are often sub-inhibitory [9] (*i.e*. too low to cause a significant growth inhibition of susceptible bacteria in culture [10]). However, sub-inhibitory concentrations might still affect the microbial community. They could slow down bacterial growth [11] and the selective pressure they exert might be sufficient to offset the biological cost of resistance, and thereby, contribute to resistance selection [12]. An increasing body of laboratory evidence with pure cultures shows that sub-inhibitory concentrations of antibiotics might select for ARB [11, 13] and induce a wide range of response mechanisms including quorum sensing, biofilm formation and the expression of genes involved in antibiotic resistance, virulence and the SOS response [14]. Furthermore, sub-inhibitory concentrations of antibiotics might select for mutants with a lower fitness cost than the ones selected at inhibitory concentrations [11] and might be sufficient for the maintenance of plasmids coding for multidrug resistance [15]. These concentrations might also stimulate ARG integration in mobile genetic elements (MGEs) and dissemination through horizontal gene transfer (HGT) [16–19]. Therefore, some antibiotics might select for resistance in a dose-independent manner and contribute to resistance development at concentrations below the minimal inhibitory concentration (MIC) of susceptible bacteria [12].

Published studies working to elucidate the effects of sub-inhibitory concentrations of antibiotics on bacterial communities and their associated resistome used culture-based approaches. These approaches deal with a reduced portion of the bacterial community under controlled growth conditions. Therefore, they do not necessarily account for the complexity of natural environments [20]. Experiments with natural microbial communities can be monitored by environmental DNA sequencing (i.e., metagenomic approaches). Metagenomic studies avoid culturing biases and provide access to a wider proportion of the environmental microbiome without altering the original conditions of the environmental matrix. Nevertheless, the genes and taxa identified using metagenomics approaches are not necessarily actively responding to the antibiotic. Thus, the combination of metagenomics with RNA-based analyses should provide a better understanding of the taxa and genes involved in the response to sub-inhibitory concentrations of antibiotics.

In this study, we used a microcosm approach to simulate in situ conditions (presence of the whole bacterial community in their environmental matrix with no addition of growth stimulants). We evaluated the response of environmental bacteria to sub-inhibitory concentrations of antibiotics in river water microcosms and established relationships between antibiotic dose and the magnitude of the response using gentamicin as a model antibiotic. Gentamicin is an aminoglycoside used in both human therapy and veterinary medicine. Residual concentrations of excreted gentamicin have been detected in wastewater treatment plants [21]. Gentamicin at sub-inhibitory concentrations has been shown to induce resistance development in pure cultures [22]. Moreover, aminoglycoside resistance genes are widely distributed in both chromosomal and plasmid class 1 integrons [23], which are considered major vectors of antibiotic resistance dissemination [24]. Thus, we identified the bacteria and genes involved in the environmental response to gentamicin at sub-inhibitory concentrations in river water microcosms using a combination of metagenomics and RNA-based analyses. Class 1 integron cassettes were sequenced to screen for aminoglycoside resistance genes. The response to sub-inhibitory concentrations of gentamicin was compared to that observed under gentamicin pollution at inhibitory concentrations. We hypothesized that inhibitory concentrations of gentamicin would induce a stronger response than sub-inhibitory concentrations but that the latter would induce shifts in the composition of bacterial communities and increase the abundance and transcription of gentamicin resistance genes in the overall community. To the best of our knowledge, this is the first study 1) showing that sub-inhibitory concentrations of gentamicin induce a response in environmental bacterial communities and their associated resistome in the aquatic environment and 2) establishing links between antibiotic dose and the magnitude of the response observed in the environment.

## Materials and methods

### Sampling and determination of gentamicin effect on river water enrichments

Rhône river water was sampled in Lyon (45°45’08.3”N 4°50’11.3”E). In order to determine which gentamicin concentrations had sub-inhibitory effects on Rhône river water, bacterial enrichments of 0.5 ml of river water in 4.5 ml of R2A medium without antibiotics or with gentamicin at 10, 50, 100, 500 or 1000 ng/ml were prepared. Immediately after mixing by inversion, 200 µl of bacterial enrichments were transferred to a 96-well culture plate and incubated for 45 h at 29 °C under continuous shaking in the MultiSkan GO Plate Reader (Thermo Scientific Scientific, Waltham, Massachusetts, USA). OD_600_ was measured every hour (Figure S1 in Supplementary Information). Based on these results, 10 and 50 ng/ml were identified as sub-inhibitory concentrations. Since both 500 ng/ml and 1 µg/ml were identified as inhibitory, an intermediary inhibitory concentration of 800 ng/ml was the one selected for this study.

### Microcosm experiment using gentamicin-polluted river water

One-liter water microcosms containing 0, 10, 50 or 800 ng/ml of gentamicin were prepared and incubated for 48 h at room temperature. Microcosms were shaken at 35 rpm to emulate river currents. Triplicates were made for each gentamicin concentration. After 0, 24- and 48-h exposure, DNA and RNA were extracted from water microcosms. Gentamicin concentration was measured for every sample and exposure time by HPLC-MS/MS, using the method described by Guironnet et al. [25] (Table S1 in Supplementary information).

### DNA extraction from water microcosms

For each extraction, 90 ml of river water were filtered through a 0.2 µm filter using a vacuum pump. Then, the bacteria on the filter were resuspended in 1 ml of CTAB Buffer (Promega, Madison, Wisconsin, USA) and heated for 5 min at 95 °C. After vortexing for 1 min, 300 µl of supernatant were added to 300 µl of Lysis Buffer (Promega) and purified using the Maxwell® RSC Instrument and the Maxwell® RSC PureFood GMO and Authentication Kit (Promega). DNA was eluted in 100 µl of Elution Buffer (Promega).

### RNA extraction from water microcosms

For each extraction, 90 ml of river water were filtered through a 0.2 µm filter using a vacuum pump. Then, the bacteria on the filter were resuspended in 1 ml of CTAB Buffer (Promega) with 2% 1-thioglycerol and vortexed for 1 min. 200 µl of supernatant were added to 200 µl of Lysis Buffer (Promega) and purified using the Maxwell® RSC Instrument and the Maxwell® RSC miRNA Tissue Kit (Promega), which includes DNase treatment. RNA was eluted in 60 µl of nuclease-free water.

### Estimation of the size of the total and active bacterial communities

The size of the total and active bacterial communities was estimated by quantifying the V3 region of the 16 S rRNA gene and of 16 S rRNA by qPCR and RT-qPCR, respectively, using the primers 341 F (5′-CCTACGGGAGGCAGCAG- 3′) and 534 R (5′-ATTACCGCGGCTGCTGGCA-3′) [26, 27]. Quantitative PCR assays were carried out using the Corbett Rotor-Gene 6000 (QIAGEN, Hilden, Germany) in a 20 µl reaction volume containing GoTaq qPCR Master Mix (Promega), 0.75 µM of each primer and 2 µl of DNA or of cDNA retrotranscribed from extracted RNA using the SuperScript III RT (Thermo Fisher Scientific). Two non-template controls were included in all the assays. Standard curves for all the assays were obtained using 10-fold serial dilutions of a linearized plasmid pGEM-T Easy Vector (10^7^–10^2^ copies) containing the 16 S rRNA gene of *Pseudomonas aeruginosa* PAO1. Cycling conditions for qPCR amplification were 95 °C for 2 min followed by 30 cycles of 95 °C for 15 s, 60 °C for 30 s and 72 °C for 30 s. Melting curves were generated after amplification by increasing the temperature from 60 °C to 95 °C. Then, two-tailed ANOVA tests and t-student tests between each group (each gentamicin concentration and each exposure time) and the average between all groups were performed using the ggpubr package in R (Figure S2 in Supplementary Information).

### 16S rRNA gene and 16S rRNA sequencing and analysis

The V3-V4 hypervariable regions of the bacterial 16S rRNA and its gene and were amplified using forward (5′-TCGTCGGCAGCGTCAGATGTGTATAAGAGACAGTCGTCGGCAGCGTCAGATGTGTATAAGAGACAGCCTACGGGNGGCWGCAG-3′) and reverse (5′-GTCTCGTGGGCTCGGAGATGTGTATAAGAGACAGGTCTCGTGGGCTCGGAGATGTGTATAAGAGACAGGACTACHVGGGTATCTAATCC-3′) [28] primers. DNA was amplified using the Titanium Taq DNA Polymerase (Takara Clontech, Kusatsu, Japan) and the following conditions: 94 °C for 3 min followed by 30 cycles of 94 °C for 30 s, 55 °C for 30 s and 72 °C for 30 s and a final extension step at 72 °C for 5 min. cDNA was amplified using the Platinum Taq DNA Polymerase (Thermo Fisher Scientific) and the same conditions as DNA. DNA and cDNA libraries were prepared from amplified products based on Illumina’s “16S Metagenomics Library Prep Guide” (15044223 Rev. B) using the Platinum Taq DNA Polymerase (Invitrogen) and the Nextera XT Index Kit V2 (Illumina, San Diego, California, USA). DNA and cDNA were sequenced using the MiSeq System and the MiSeq Reagent Kit v2 (Illumina). Reads were trimmed to meet a quality score of Q20. Then, pair-ended reads were assembled using PANDAseq [29] at a sequence length between 410 and 500 bp and an overlap length between 20 and 100 bp using the rdp_mle algorithm. Finally, each of the DNA sequences was annotated to the genus level using the Ribosome Data Project (RDP) database and the RDP Bayesian classifier [30] using an assignment confidence cut-off of 0.6. The genera that had less than 10 associated sequences in the ensemble of DNA or cDNA sequences were removed. Finally, the relative abundances of the remaining genera were assessed and normalized per number of copies of the 16 S rRNA gene or per l of water.

### Sequencing of pre-clinical class 1 integron cassettes

Since aminoglycoside resistance genes are often integrated in class 1 integrons, pre-clinical class 1 integron cassettes, which are often found in the environment, were amplified and sequenced in order to identify the ARGs that they encoded. Specific primers for the integrase *int1* (HS463a: 5′-CTGGATTTCGATCACGGCACG-3′ and HS464: 5′- ACATGCGTGTAAATCATCGTCG-3′) [31] were used to detect class 1 integrons in water DNA samples. Three µl of DNA were amplified using the Platinum Taq DNA Polymerase (Thermo Fisher Scientific) as follows: 94 °C for 2 min followed by 35 cycles of 94 °C for 30 s, 60 °C for 30 s and 72 °C for 90 s and a final extension step at 72 °C for 5 min. All water DNA samples contained class 1 integrons. Then, pre-clinical integron gene cassette arrays were amplified from water DNA using MRG284 (5’- GTTACGCCGTGGGTCGATG-3’) and MRG285 (5′- CCAGAGCAGCCGTAGAGC-3′) primers [32]. Annealing was performed at 65 °C for 2 min instead of 60 °C for 30 s. Then, amplicons were cleaned-up using AMPure XP beads (Beckman-Coulter, Brea, California, USA) and sequenced using the Nextera XT Library Prep Kit and Indexes (Illumina) according to the Illumina’s “Nextera XT DNA Library Prep Kit” reference guide (15031942 v03). DNA sequencing was performed using the MiSeq System and the MiSeq Reagent Kit v2 (Illumina).

Metagenomics reads were trimmed using the Fastq Quality Trimmer tool of the FASTX-Toolkit. Nucleotides that did not meet a minimum quality score of Q20 were trimmed from the sequences and sequences shorter than 100 nucleotides after trimming were removed. Forward and reverse reads were blasted separately against the CARD database using Diamond [33]. The obtained results were filtered at a minimum identity of 90%, a minimum length of 50 amino acids and an e-value of 10^−10^. The best hit was used. Results obtained from blasting forward and reverse read were identical. Therefore, the analysis was continued using only forward reads. The abundance of the aminoglycoside resistance genes encoded by class 1 integrons was normalized by sequencing depth (Figure S3 in Supplementary Information).

### Quantification of aminoglycoside resistance gene abundance and transcription

Thirty-two aminoglycoside resistance genes coding for aadA aminoglycoside nucleotidyltransferases and AAC(6′) aminoglycoside acetyltransferases were identified in integron cassette arrays. Since abundance calculations of aminoglycoside resistance genes based on the sequencing of amplified integron cassettes lack accuracy, primer pairs were designed to target these genes by qPCR/RT-qPCR. The high similarity of the sequences of genes from the same family constrained accurate assignation of reads to all different genes within a gene family (i. e. reads that were identified as one of the genes could be another gene from the same family). In addition, this high similarity hindered the design of specific qPCR primer pairs for each single gene within the same family. Therefore, two primer pairs were designed to target conserved regions of the identified *aadA* (17 genes) and the 15 *aac(6’)* genes, respectively. Information showing primer sequences and targeted genes are shown in Table S2 (Supplementary Information). Then, *aadA* and *aac(6*′*)* genes were quantified by qPCR in water DNA and cDNA samples in order to evaluate the abundance and transcription of aminoglycoside resistance genes under gentamicin pollution at different concentrations and exposure times. The Corbett Rotor-Gene 6000 (QIAGEN) amplified target genes in a 20 µl reaction volume containing GoTaq qPCR Master Mix (Promega), 0.75 µM of each primer and 2 µl of DNA or cDNA. Two non-template controls were also included in all the assays and non-retrotranscribed controls were included in cDNA amplifications. Standards for qPCR assays were obtained from water DNA samples using *aadA* and *aac(6’)* primers. Standards were cloned and transformed using the TOPO TA cloning Kit (Thermo Fisher Scientific) and normalized to 10^8^ copies/µl. Standard curves were made using 10-fold serial dilutions of the standards (10^7^–10^2^ copies/µl). Cycling conditions for qPCR amplification were 95 °C for 2 min followed by 35 cycles of 95 °C for 30 s, 55 °C for 30 s and 72 °C for 30 s. Melting curves were generated after amplification by increasing the temperature from 60 °C to 95 °C. The number of copies per µl of qPCR reaction obtained from the amplification of *aadA* and *aac(6’)* genes or transcripts were normalized by the copies of the 16S rRNA gene or transcripts per µl of qPCR reaction to assess their relative abundance in water. Average values and the percentage of standard deviation calculated for each condition are shown in Table S3 (Supplementary Information). Since gentamicin pollution at 800 ng/ml provoked a higher increase of gene abundance and transcription than sub-inhibitory concentrations, dose–response graphs were created only for sub-inhibitory concentrations for better visualization of the effect of gentamicin pollution at sub-inhibitory concentrations on aminoglycoside resistance gene abundance and transcription. Two-tailed ANOVA tests were performed using the ggpubr package in R.

### Metagenomics sequencing (short and long reads) and hybrid assembly

Short-read metagenomics libraries were prepared from ≤1 ng of DNA obtained from water microcosms using the Nextera XT Library Prep Kit and Indexes (Illumina), as detailed in Illumina’s “Nextera XT DNA Library Prep Kit” reference guide (15031942 v03). DNA sequencing was performed using the MiSeq System and the MiSeq Reagent Kit v2 (Illumina).

Triplicates from microcosms polluted at 50 ng/ml of gentamicin after 0, 1 or two-day exposure were pooled. Then, pooled DNA ( ≤ 100 ng) was sequenced using the MiniON, the flow cell R 9.4.1 and the Ligation Sequencing Kit SQK-LSK109 (Oxford Nanopore Technologies, Oxford, UK). Long-read metagenomics libraries were prepared as detailed in Oxford Nanopore’s “1D Genomic DNA by Ligation” protocol.

Short-reads sequenced using Illumina’s MiSeq System and filtered according to the criteria described by Minoche et al. [34] were co-assembled to long-reads obtained from the Oxford Nanopore sequencing using Unicycler [35]. Two assemblies were done in parallel: the first one included all sequences from microcosms polluted at 50 ng/ml of gentamicin, regardless of exposure time, and the second one included only sequences from microcosms polluted at 50 ng/ml of gentamicin after two-day exposure. Then, reads were mapped onto the contigs using Bowtie2 (ref. [36]) to generate BAM files. Data regarding the hybrid assembly of short and long reads can be found in Table S4 (Supplementary Information). Profiles were created for each individual sample and merged using the anvi’o [37] metagenomic workflow. The assembled contigs were blasted against the CARD antibiotic gene database [38] to identify ARGs. Results were filtered at an amino acid identity percentage of 60%, 33 amino acid length and an e-value of 10^−5^. The best hit was used. Finally, contigs were binned based on their differential coverage across samples using anvi’o [37], and the bins were refined based on differential coverage and sequence composition. Bins with <50% completion and ≥10% redundancy were discarded, since they were considered low-quality metagenome assembled genome (MAG) drafts according to Bowers et al. [39]. The taxonomy of the bins estimated from the ORFs using kaiju [40] (as part of the anvi’o workflow) was confirmed by “blasting” all contigs included in each bin against the RDP database to identify ribosomal sequences.

## Results

### Gentamicin effect on overall growth of river water bacteria in bacterial enrichments and microcosms

The effects of a range of gentamicin concentrations from 0 ng/ml to 1 µg/ml on total water bacteria enriched in growth medium were assessed (Figure S1 in Supplementary Information). Although both 10 and 50 ng/ml of gentamicin delayed the onset of the growth curve and slower growth rates were observed in enriched water bacteria exposed to 50 ng/ml, there was no significant overall growth inhibition after 45-hour exposure to either of these two concentrations compared to non-polluted bacterial enrichments. On the other hand, concentrations from 100 ng/ml and above induced a significant overall growth inhibition (*p* values <0.05) in water bacterial enrichments over the 45-hour incubation. No growth was observed in enriched media polluted with gentamicin at 500 ng/ml nor at 1 µg/ml during the 45-hour incubation. Therefore, a concentration of 800 ng/ml of gentamicin was chosen as an inhibitory concentration control for future experiments; 10 ng/ml and 50 ng/ml were the sub-inhibitory concentrations used to pollute river water microcosms.

In the river water microcosms (with no added organic substrate), 800 ng/ml of gentamicin significantly reduced both the abundance and the activity of the river water bacterial community after a two-day exposure (*p* values <0.05) and was, therefore, considered to be inhibitory to river water bacteria (Figure S2 in Supplementary Information) as observed in bacterial enrichments. The sub-inhibitory concentrations, 10 and 50 ng/ml, did not have any significant effect on growth or activity in the river water microcosms after a 2-day exposure. Both the abundance and the activity of river water bacterial communities increased over time in non-polluted control microcosms and those polluted with gentamicin at 10 and 50 ng/ml. Therefore, these concentrations were sub-inhibitory to river water bacterial communities both in vitro enrichments and in microcosms.

### Gentamicin at 50 ng/ml increased the abundance of *Limnohabitans* in both total and active bacterial communities from river water microcosms after only one day of exposure

**Fig. 1:**
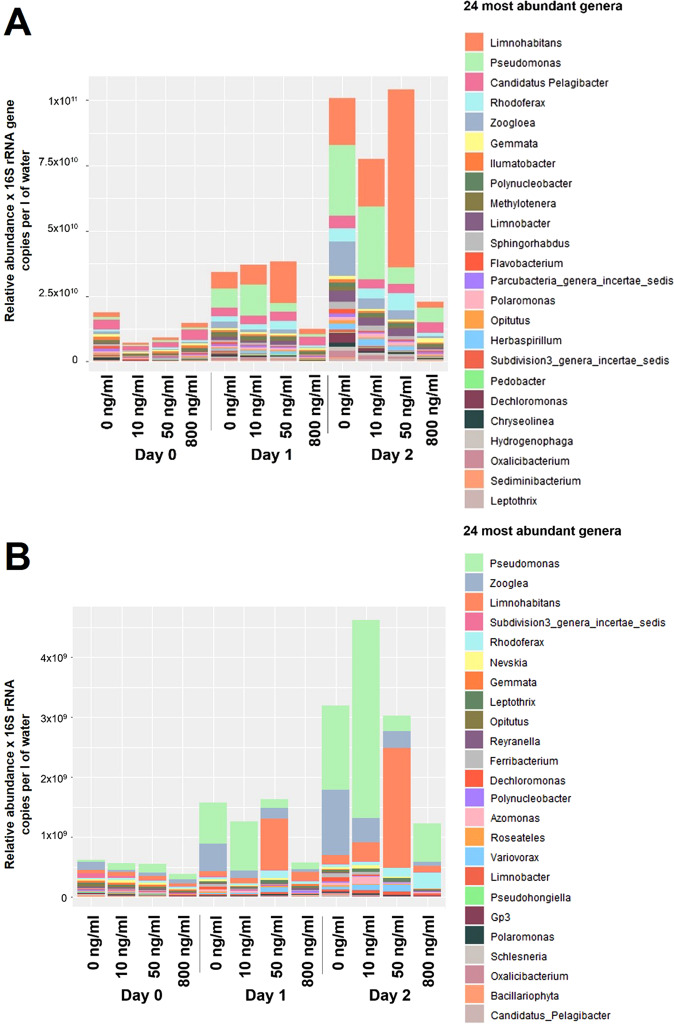
Changes in the inferred absolute abundance and activity of the 24 most abundant genera in river water microcosms. **A** Average genus relative abundance obtained from the sequencing of the 16S rRNA gene, normalized per number of copies of the 16S rRNA gene per l of water. **B** Average genus relative abundance obtained from the sequencing of the 16S rRNA (cDNA), normalized per 16S rRNA copies per l of water. Obtained from non-polluted water microcosms and microcosms polluted at 10, 50 or 800 ng/ml of gentamicin after 0, 1 or 2-day exposure. *n* = 3.

All gentamicin concentrations tested affected the composition of total and active communities in the river water microcosms. First, the abundance of several genera, including *Zooglea, Sphingohabdus* and *Polynucleobacter*, was slightly lower in the total and active communities from microcosms polluted at 10 ng/ml of gentamicin after a 2-day exposure compared to non-polluted controls (Fig. 1). In addition, the abundance of *Limnohabitans* and *Pseudomonas* transcripts increased over time at this gentamicin concentration. However, this concentration did not have a strong impact on community composition. In microcosms polluted at 50 ng/ml of gentamicin, a shift in community composition was observed after a 1-day exposure with a concomitant increase in the abundance of *Limnohabitans* in both total and active communities. This increase continued after two days of exposure, when *Limnohabitans* dominated the communities present in river water microcosms (58.58% ± 12.51% of the total community and 60.36% ± 19.35% of the active community). Other genera, such as *Pseudomonas* and *Zooglea*, were less abundant in microcosms polluted at 50 ng/ml of gentamicin after a two-day exposure than in non-polluted controls. On the other hand, at 800 ng/ml, a concentration that inhibited overall bacterial growth and activity in water microcosms, the abundance of virtually all genera present in both total and active communities was reduced compared to the microcosms at lower gentamicin concentrations. However, *Pseudomonas* increased its abundance and transcription over time and seemed to be able to grow at gentamicin concentrations of 800 ng/ml. In addition, even though *Rhodoferax* abundance was lower in microcosms with 800 ng/ml of gentamicin than in the other microcosms, its transcription levels increased over time (Fig. 1).

### Gentamicin at 50 ng/ml increased the abundance and transcription of gentamicin resistance genes from the aac(6’) family present in class 1 integron cassettes

Aminoglycoside resistance genes were found in sequenced class 1 integrons cassettes and belonged to two main families: the *aadA* genes coding for aminoglycoside nucleotidyltransferases, which confer resistance to spectinomycin and streptomycin [41], and the *aac(6’)* genes coding for *AAC*(6’) acetyltransferases, which mediate resistance to several aminoglycosides including gentamicin [41] (Figure S3 in Supplementary Information). Seventeen *aadA* genes and fifteen *aac(6’)* genes were found in class 1 integrons. While the relative abundance of the total number of *aadA* gene-related reads found within the class 1 integrons generally remained constant at different gentamicin concentrations and exposure times, the relative abundance of *aac(6’)* gene-related reads found within the class 1 integrons increased in gentamicin-contaminated river water microcosms over time.

**Fig. 2:**
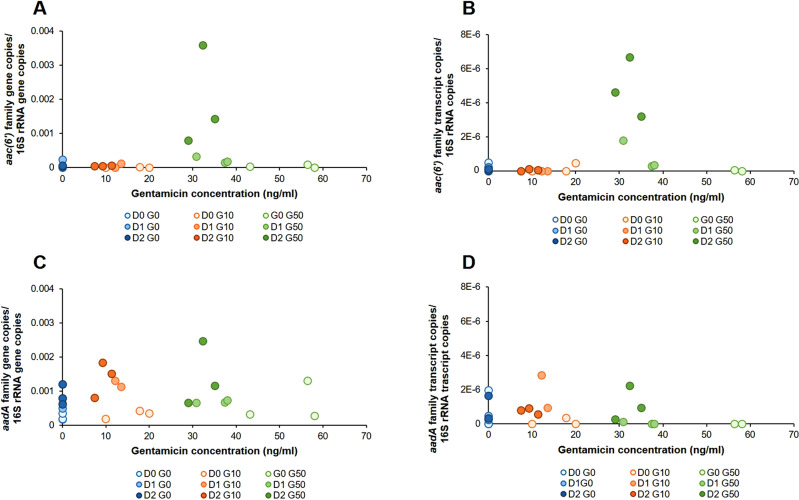
Dose-response linking sub-inhibitory gentamicin concentrations and relative abundance of aminoglycoside resistance genes and transcripts. **A** Relative abundance of *aac(6’)* family gene copies; **B** relative abundance of *aac(6’)* family transcript copies; **C** relative abundance of *aadA* family gene copies; **D** relative abundance of *aadA* family transcript copies. Blue: 0 ng/ml of gentamicin. Orange: 10 ng/ml of gentamicin. Green: 50 ng/ml of gentamicin. Dot colour gets darker with exposure time (DO; day 0, D1: day 1; D2; day 2). *aac(6’)* family gene primers: qPCR efficiency = 0.99; *R*^2^ linearity coefficient = 0.997. *aadA* family gene primers: qPCR efficiency = 1; *R*^2^ linearity coefficient = 0.996. ANOVA degrees of freedom: 8. ANOVA F-values: 4.88 (*aac(6’)* abundance); 17.25 (*aac(6’)* transcription); 2.52 (*aadA* abundance); 0.98 (*aadA* transcription). *n* = 3.

To avoid potential quantitative metagenomic biases, the abundance of these genes and their transcripts was determined by qPCR. Whereas in non-polluted controls and in microcosms polluted at 10 ng/ml of gentamicin, the relative abundance (normalized by bacterial biomass) of *aac(6*′*)* genes and transcripts remained stable over time, both increased over exposure time in microcosms polluted at 50 ng/ml of gentamicin (Fig. 2). After a two-day exposure, the average relative abundance of *aac(6*′*)* genes and transcripts in microcosms contaminated at 50 ng/ml was two orders of magnitude higher than that observed in non-polluted controls and samples contaminated at 10 ng/ml of gentamicin (ANOVA *p* values of 0.0025 and 5.7 × 10^−7^, respectively). The quantity of *aac(6’)* genes and transcripts normalized per water volume (one liter of water) also increased 38 and 60-fold, respectively, under gentamicin pollution at 50 ng/ml after two days (Table S3a in Supplementary Information). On the other hand, at 800 ng/ml, this increase was higher, 277-fold for genes and 952-fold for transcripts (Table S3a in Supplementary Information). Gentamicin concentration decreased over the 2-day exposure in all polluted microcosms (Table S1 in Supplementary Information). The relative abundance of *aac(6’)* genes and transcripts after the 2-day exposure was between 10 and 100 times higher at 800 ng/ml of gentamicin than at 50 ng/ml (Table S3b in Supplementary Information), whereas the difference in the number of copies of *aac(6’)* genes and transcripts per liter of water was lower, approximately seven and 15 times more genes and transcripts, respectively, at 800 ng/ml than at 50 ng/ml of gentamicin (Table S3a in Supplementary Information). Both conditions had significantly more *aac(6*′*)* genes and transcripts than the 0 and 10 ng/ml conditions. The relative abundance of *aadA* genes and transcripts remained relatively constant over exposure time and gentamicin concentration, both normalized by bacterial biomass and by water volume (Tables S3a and S3b in Supplementary Information). A slightly higher abundance of *aadA* genes was observed over time at 10 and 50 ng/ml of gentamicin than in non-polluted controls (ANOVA *p* value of 0.049), but this was not followed by a similar increase in transcription levels (ANOVA *p* value of 0.48).

### A gentamicin resistance gene was found in a partial genome reconstruction from *Limnohabitans*

**Fig. 3:**
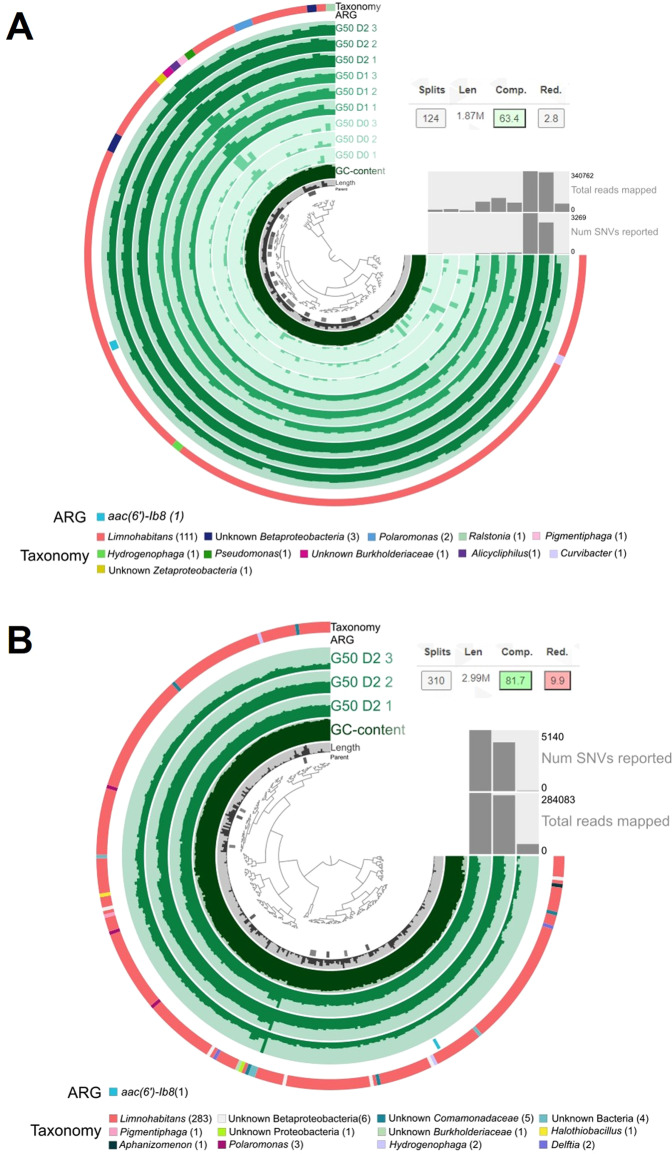
*Limnohabitans* MAGs containing a gentamicin resistance gene obtained from the hybrid co-assembly short and long of metagenomic sequences from water microcosms polluted at 50 ng/ml of gentamicin. **A** Samples exposed to gentamicin for 0, 1 and 2 days; **B** Samples exposed to gentamicin for 2 days. Taxonomy shown is based on Open Reading Frames (ORFs) on contigs search against the protein reference database kaiju.

A partial metagenome assembled genome (MAG) identified as *Limnohabitans* was reconstructed from the hybrid assemblies of short and long metagenomics reads from microcosms polluted at 50 ng/ml of gentamicin at all exposure times (Fig. 3A) and after a two-day exposure (Fig. 3B). The *Limnohabitans* bins obtained from both assemblies contained a sequence that matched a gene coding for an aminoglycoside acetyltransferase involved in gentamicin resistance, *aac(6’)-Ib8*, with an identity of 67.6% over 34 amino acids. No matches were found for genes coding for aadA aminoglycoside nucleotidyltransferases. The bin reconstructed from sequences from microcosms with gentamicin at 50 ng/ml after 0, 1 and two-day exposures had a completion of 63.4%, a redundancy of 2.8%, 107 contigs, a length of 1.87 Mbp and a mean coverage across samples of 2.2x (Fig. 3A), whereas the one reconstructed from sequences after two-day exposure had a completion of 81.7%, a redundancy of 9.9%, 300 contigs, a length of 2.99 Mbp and a mean coverage across samples of 5.68x (Fig. 3B). All contigs from each of the two bins were blasted against the RDP database to identify ribosomal sequences. In both cases, only one contig matched the RDP database. It was identified in both bins as *Limnohabitans*, with 99.58% identity over 1 411 nucleotides.

## Discussion

The aim of this study was to determine whether sub-inhibitory concentrations of gentamicin induced a response in environmental bacterial communities in their environmental matrix and to evaluate the magnitude of that response as a function of antibiotic concentration. An increasing body of evidence suggests that sub-inhibitory concentrations of antibiotics might stimulate changes in the bacterial communities and their associated resistome and the links between antibiotic dose and community response in environmental bacteria need to be elucidated [4, 42].

This research shows that sub-inhibitory concentrations of gentamicin may have an environmental impact. Gentamicin at overall sub-inhibitory concentrations possibly favors different bacteria than at inhibitory concentrations due to differences in resistance levels and mechanisms. For example, *Pseudomonas*, which dominated at inhibitory concentrations, might not be as competitive at sub-inhibitory concentrations at which *Limnohabitans* dominated. Thus, *Limnohabitans* would dominate at intermediary concentrations by taking advantage of the possible inhibition of some competitors for nutrients. This is consistent with overall growth remaining high at these “sub-inhibitory” concentrations, although some members of the community were clearly not favored. Therefore, the use of the term “sub-inhibitory”, which has been defined in pure cultures, should not be extrapolated to complex bacterial communities in their natural setting, where the effects of antibiotics may vary from what is observed in pure cultures in vitro [43].

In addition, by analyzing class 1 integrons cassettes, we were able to link gentamicin concentration to community response through the increase in the abundance and transcription of the *aac(6’)* acetyltransferase gene family identified in these cassettes. The relative abundance and transcription levels of *aac(6’)* genes were lower at sub-inhibitory concentrations than in microcosms with 800 ng/ml of gentamicin (Table S3b in Supplementary Information). However, bacterial biomass was significantly reduced in microcosms at this inhibitory concentration (Figure S2b in Supplementary Information), and this increase in gene abundance and transcription was lower when normalized by water volume than when normalized by bacterial biomass (Figures S2a and S2b in Supplementary Information). Therefore, the selective pressure imposed by inhibitory concentrations of gentamicin inhibited most of the members of the community and resulted in a higher abundance of gentamicin resistance genes per 16S rRNA gene, whereas gentamicin at 50 ng/ml selected for *aac(6’)* genes in water microcosms without inhibiting overall bacterial growth. Thus, gentamicin at sub-inhibitory concentrations may select for gentamicin resistance genes in the environment and contribute to the burden of antibiotic resistance. In addition, the slightly higher abundances of *aadA* family genes in the presence of gentamicin could be a consequence of co-selection of these genes with *aac(6’)* genes located in the same integron cassettes. Unlike the *aac(6’)* family genes, the *aadA* family genes (which are often found in class 1 integrons) do not confer resistance to gentamicin [44], and although they were not targeted individually by qPCR/RT-qPCR, none of them were found in the hybrid assembly of short (Illumina MiSeq) and long (Oxford Nanopore) metagenomic reads. Altogether, our results did not show that *aadA* genes were involved in an active response to gentamicin addition.

Finally, a *Limnohabitans* MAG obtained from the hybrid assembly of short and long metagenomic reads putatively contained a gentamicin resistance gene (*aac(6’)-Ib8*). This gene was originally found in a human pathogen, *Enterobacter cloacae* [45] and has not been previously associated with *Limnohabitans*. The presence of this gentamicin resistance gene in the *Limnohabitans* genome could explain in part its dominance at measurable, but generally overall sub-inhibitory, gentamicin concentrations. This supports the hypothesis that *Limnohabitans* can actively respond to gentamicin, survive at sub-inhibitory concentrations and dominate more sensitive bacteria. If this gene could explain *Limnohabitans* lack of inhibition at 50 ng of gentamicin/ml, the lack of *Limnohabitans* resistance at 800 ng/ml might be due the inefficiency of this gene to protect at high concentrations.

In conclusion, this study was, to the best of our knowledge, the first to show that sub-inhibitory concentrations of antibiotics induced a response in environmental bacteria and their associated resistome in river water microcosms simulating in situ conditions and to establish relationships between antibiotic dose and the selective force for aminoglycoside resistance genes. These results support the concern that sub-inhibitory concentrations may have a selective potential in the environment [46, 47]. Therefore, in order to improve the accuracy of risk assessments, efforts should be focused on assessing antibiotics’ potential to induce resistance selection in environmental bacteria in situ regardless of the effects of antibiotics on overall growth inhibition in complex communities. In addition, the genes that increased their abundance and transcription at sub-inhibitory gentamicin concentrations were present in class 1 integrons, which are active in the dissemination of antibiotic resistance among environmental bacteria and to human and animal pathogens [48–50]. Thus, gentamicin resistance genes selected under gentamicin pollution at sub-inhibitory concentrations could be mobilized and disseminated across the environmental microbiome and into the human and animal microbiomes.

The aim of this study was to evaluate the selective potential of sub-inhibitory concentrations of antibiotics in the environment. Therefore, the dissemination potential of environmental ARGs is out of the scope of this study. Further studies should determine the risk of dissemination to the human and animal microbiomes of ARGs present in the environment that are selected for at sub-inhibitory concentrations of antibiotics. In addition, this study was based on a single environmental model and a single antibiotic. Future studies should compare the response of different environments to the same antibiotic and establish patterns between the composition of the environmental matrix and/or environmental conditions and the observed response. Furthermore, a combination of antibiotics might reflect more accurately the situation occurring in freshwater environments receiving treated wastewater [51], and the combination of antibiotics at sub-inhibitory concentrations might increase resistance selection [52]. Therefore, studies analyzing the effect of combined antibiotics at sub-inhibitory concentrations on the environmental microbiome and resistome are still needed to fill knowledge gaps and provide a more realistic risk assessment. Finally, this study evaluated a short-term environmental response to gentamicin, but did not provide any information concerning the response at longer exposure times nor the persistence of resistance in the absence of antibiotic pressure. These elements are key to a more thorough understanding of the risk posed by sub-inhibitory concentrations of antibiotics and should be evaluated in the future.

## Supplementary information

Supplement information

## Data Availability

The datasets generated and analyzed during the current study are publicly available in the Environmental Microbial Genomics Group repository: https://www.mmnt.net/db/0/0/ftp.ec-lyon.fr/pub/ADN/
