# Supplementary material for: Gentamicin at sub-inhibitory concentrations selects for antibiotic resistance in the environment
Source: ISME Commun. 2022 Mar 30;2:29. doi: 10.1038/s43705-022-00101-y (PMC9723587; doi:10.1038/s43705-022-00101-y)
Supplement: Supplementary file 1 — Supplement information [file 43705_2022_101_MOESM1_ESM.docx]

**Supplementary Information**

**Figure S1. Average OD_600_ of bacterial enrichments in R2A medium with different gentamicin concentrations measured every hour for 45 hours (left) and measured after 45 hours (right).** 150 µl of culture were transferred to a multi-well plate and incubated at 29°C for 45 hours, measuring the OD_600_ every hour. n=3.


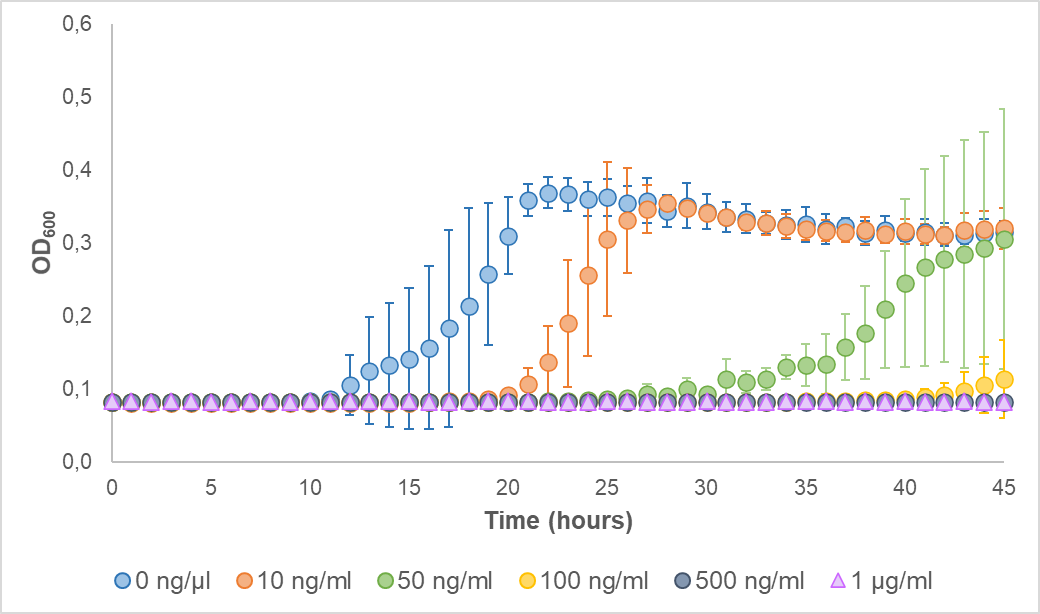

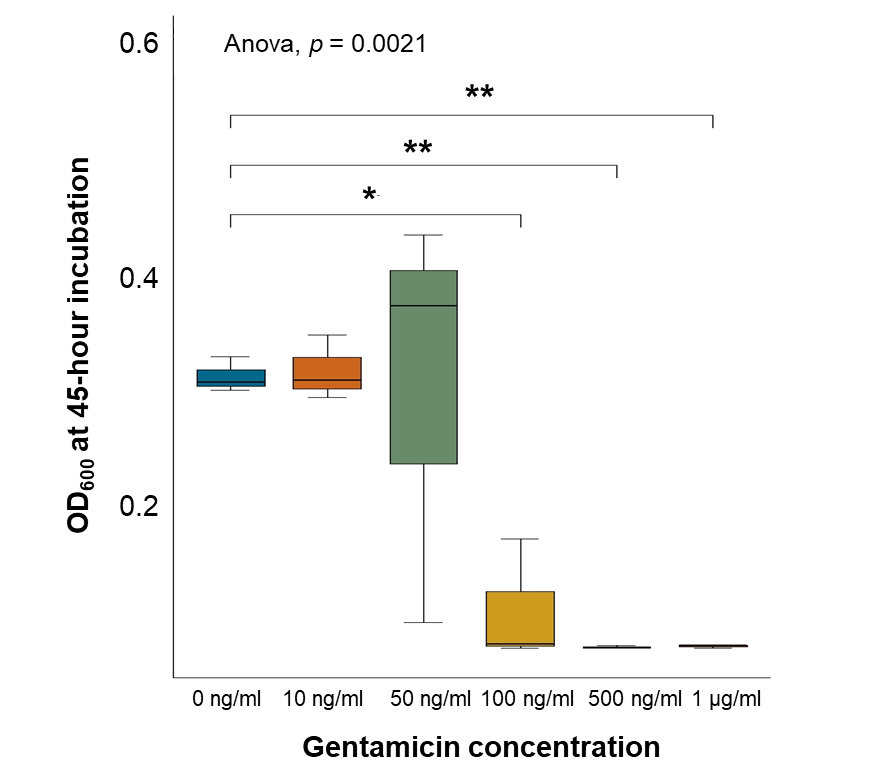


**Figure S2. Bacterial dynamics over time at different gentamicin concentrations in river water microcosms.** Copies of the 16S rRNA gene (left) and the 16S rRNA (right) per of water obtained from non-polluted water or water polluted with gentamicin at 10, 50 or 800 ng/ml after 0, 1 or 2-day exposure. qPCR efficiency=1.07. R^2^ linearity coefficient=0.996. Significant differences between each group (each gentamicin concentration and each exposure time) and the average between all groups (horizontal dashed line) were determined by a t-test. **p*-value ≤0.05. ***p*-value≤0.01. ****p*-value ≤0.001. *****p*-value≤0.0001. n=3.


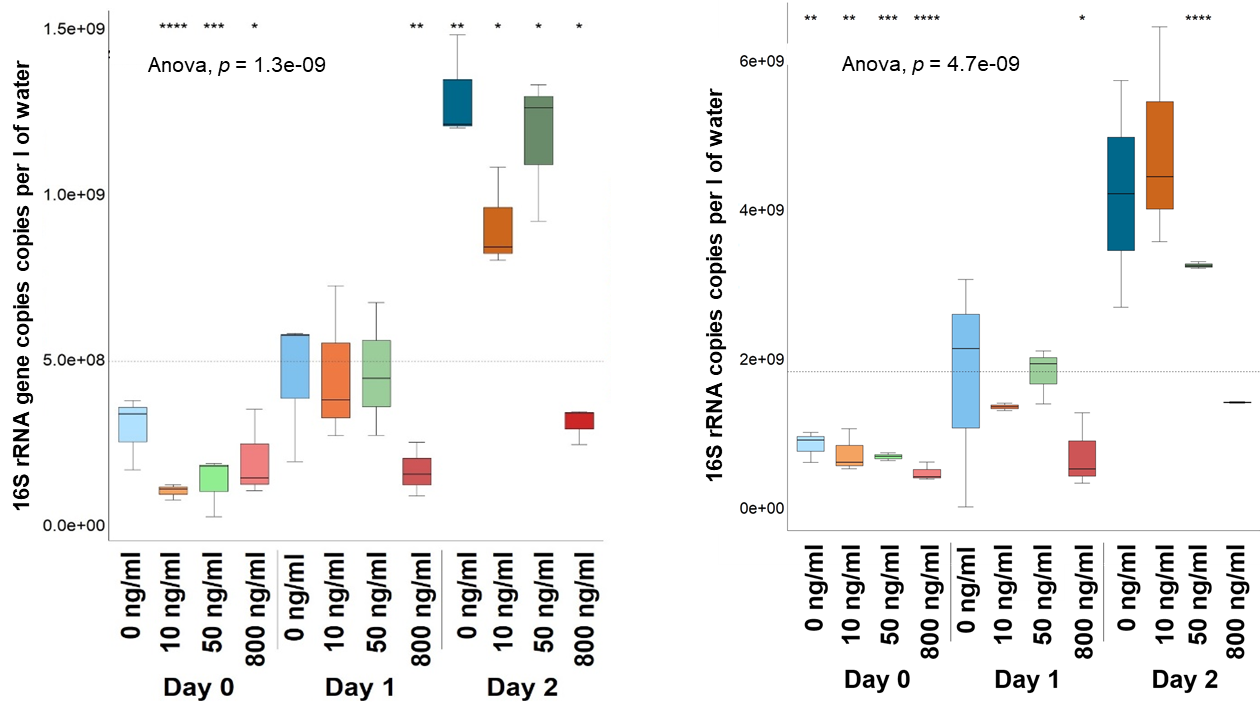


**Figure S3. Relative abundance of the aminoglycoside resistance genes from the aadA family (green) and the AAC(6’) family (orange) in class 1 integrons.** Pre-clinical class 1 integrons were amplified using MRG284/MRG285 primers and sequenced. Aminoglycoside resistance genes were identified by blasting integron reads against the CARD database using Diamond and filtered at a minimum identity of 90%, a minimum length of 50 aminoacids and an e-value of 10^-10^. The best hit was taken. Reads from genes belonging to the aadA aminoglycoside nucleotidyltransferase family or the AAC aminoglycoside acetyltransferase family, respectively, were grouped together. Then, their abundance was normalized by sequencing depth. n=3.


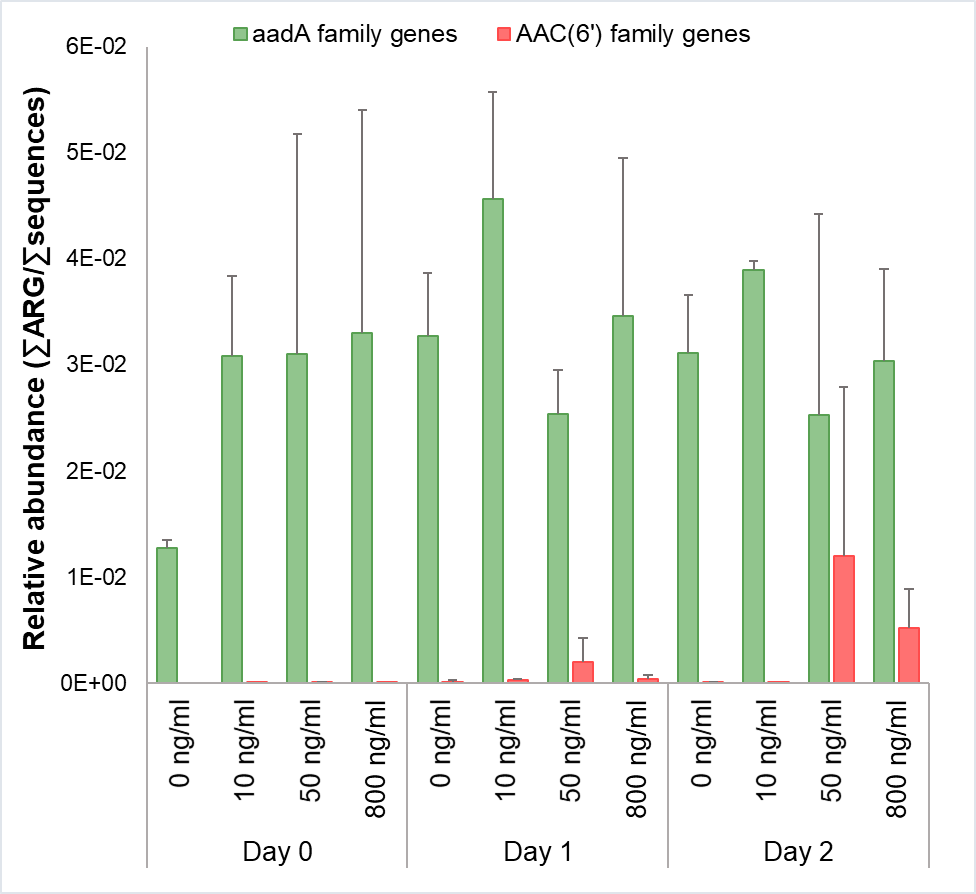


**Table S1. Gentamicin concentrations in water microcosms measured by HPLC-MS/MS.** QL: quantification limit. n=3.


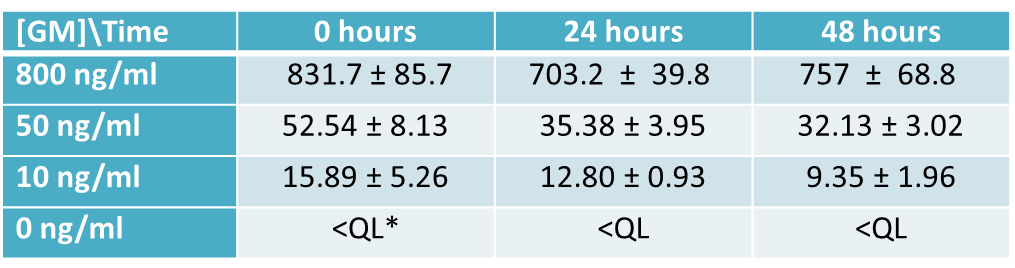


**Table S2. Details of qPCR primers targeting aminoglycoside resistance genes designed in this study.**

| **Primer pair** | **Sequence** | **Target genes** | **Amplicon size (bp)** |
| --- | --- | --- | --- |
| ***aac(6’)* family genes** | F: 5’-CATGACCTTGCGATGCTCTATG-3’ | *aac(6’)-Ib10, aac (6’)-Ib11, aac(6’)-Ib3,*  *aac(6’)-Ib4, aac (6’)-Ib7, aac(6’)-Ib8,*  *aac(6’)-Ib9, aac(6’)-Ib’, aac(6’)-Ib-cr,*  *aac(6’)-Ib,ant(3”)-II/ aac(6’)-IId,*  *aac(3)-Ib/ aac(6’)-IIb”, aac(6’)-30/ aac(6’)-Ib’, aac(6’)-Ib-Hangzhou, aac(6’)-Ib-Suzhou* | 201 |
|  | R: 5’-TCCAAGAGCAACGTACGACTG-3’ |  |  |
| ***aadA* family genes** | F: 5’-GGCCTGAAGCCATACAGTGA-3’ | *aadA3, aadA8, aadA21, aadA15, aadA24, aadA12, aadA17, aadA25, aadA23, aadA, aadA11, aadA10, aadA6/aadA10, aadA6, aadA13, aadA22, aadA2* | 234 |
|  | R: 5’-AAGAATGTCCTTACGCTGCCA-3’ |  |  |

**Table S3a. Average number of copies of the genes and transcripts from the *aac(6’)* and the *aadA* families per L of river water, and percentage of standard deviation.** The abundances of aminoglycoside resistance genes and their transcripts were assessed by qPCR normalized by volume of river water*. aac(6’)* family gene primers: qPCR efficiency=0.99, qPCR linearity=0.997. *aadA* family gene primers: qPCR efficiency=1, qPCR linearity=0.996. n=3.

|  | | *aac(6’)* family genes | | | | *aadA* family genes | | | |
| --- | --- | --- | --- | --- | --- | --- | --- | --- | --- |
|  |  | **Gene copies per L of water** | | **Transcript copies per L of water** | | **Gene copies per L of water** | | **Transcript copies per L of water** | |
|  |  | **Average** | **%SD** | **Average** | **%SD** | **Average** | **%SD** | **Average** | **%SD** |
| Day 0 | **0 ng/ml** | 10,185 | 31 | 3,000 | 62 | 1,222 | 9 | 4,444 | 173 |
|  | **10 ng/ml** | 1,111 | 100 | 889 | 173 | 626 | 39 | 778 | 173 |
|  | **50 ng/ml** | 3,519 | 78 | 1,111 | 148 | 926 | 26 | 0 | NA |
|  | **800 ng/ml** | 8,333 | 90 | 1,778 | 173 | 900 | 36 | 1,333 | 100 |
| Day 1 | **0 ng/ml** | 23,889 | 86 | 889 | 173 | 7,541 | 75 | 10,111 | 54 |
|  | **10 ng/ml** | 22,778 | 98 | 0 | NA | 8,233 | 14 | 19,778 | 97 |
|  | **50 ng/ml** | 116,481 | 84 | 17,000 | 109 | 6,319 | 40 | 889 | 173 |
|  | **800 ng/ml** | 29,259 | 65 | 5,556 | 134 | 1,807 | 6 | 0 | NA |
| Day 2 | **0 ng/ml** | 62,037 | 39 | 2,889 | 144 | 23,070 | 48 | 38,000 | 151 |
|  | **10 ng/ml** | 48,889 | 11 | 3,778 | 112 | 24,059 | 28 | 42,111 | 50 |
|  | **50 ng/ml** | 2,362,222 | 89 | 174,889 | 37 | 34,511 | 79 | 41,667 | 88 |
|  | **800 ng/ml** | 17,225,000 | 133 | 2,750,667 | 116 | 52,674 | 68 | 63,667 | 122 |

**Table S3b. Average relative abundance and percentage of standard deviation of the relative abundance of gene copies and transcripts from the *aac(6’)* and the *aadA* families.** The abundances of aminoglycoside resistance genes and their transcripts were assessed by qPCR normalized by the number of copies of the 16S rRNA gene and 16S rRNA, respectively*. aac(6’)* family gene primers: qPCR efficiency=0.99, qPCR linearity=0.997. *aadA* family gene primers: qPCR efficiency=1, qPCR linearity=0.996. n=3.

|  | | *aac(6’)* family genes | | | | *aadA* family genes | | | |
| --- | --- | --- | --- | --- | --- | --- | --- | --- | --- |
|  |  | **Gene copies/16S rRNA gene copies** | | **Transcript copies/16S rRNA copies** | | **Gene copies/16S rRNA gene copies** | | **Transcript copies/16S rRNA copies** | |
|  |  | **Average** | **%SD** | **Average** | **%SD** | **Average** | **%SD** | **Average** | **%SD** |
| Day 0 | **0 ng/ml** | 3.6E-05 | 10 | 3.1E-07 | 52 | 2.3E-04 | 41 | 6.6E-07 | 173 |
|  | **10 ng/ml** | 9.9E-06 | 104 | 1.5E-07 | 173 | 3.2E-04 | 38 | 1.2E-07 | 173 |
|  | **50 ng/ml** | 4.5E-05 | 89 | 2.4E-08 | 141 | 6.3E-04 | 92 | 0 | NA |
|  | **800 ng/ml** | 3.8E-05 | 37 | 2.6E-07 | 173 | 2.6E-04 | 26 | 3.1E-07 | 102 |
| Day 1 | **0 ng/ml** | 9.5E-05 | 132 | 2.6E-08 | 173 | 8.3E-04 | 42 | 2.3E-05 | 170 |
|  | **10 ng/ml** | 5.0E-05 | 120 | 0 | NA | 1.0E-03 | 33 | 1.9E-06 | 70 |
|  | **50 ng/ml** | 2.2E-04 | 44 | 8.2E-07 | 102 | 6.9E-04 | 6 | 4.2E-08 | 173 |
|  | **800 ng/ml** | 2.5E-04 | 109 | 8.8E-07 | 154 | 6.7E-04 | 52 | 0 | NA |
| Day 2 | **0 ng/ml** | 4.7E-05 | 27 | 7.7E-08 | 79 | 8.7E-04 | 34 | 9.8E-07 | 93 |
|  | **10 ng/ml** | 5.5E-05 | 16 | 6.4E-08 | 92 | 1.4E-03 | 38 | 7.5E-07 | 22 |
|  | **50 ng/ml** | 1.9E-03 | 75 | 4.8E-06 | 36 | 1.4E-03 | 65 | 1.1E-06 | 87 |
|  | **800 ng/ml** | 7.6E-02 | 97 | 2.6E-04 | 75 | 8.0E-03 | 60 | 5.8E-06 | 95 |

**Table S4. Hybrid assembly of Oxford Nanopore and Illumina Miseq metagenomic reads microcosms polluted with gentamicin at 50 ng/ml at all exposure times and after 2-day exposure.**

|  |  | All exposure times | 2-day exposure |
| --- | --- | --- | --- |
| Co-assembly of Illumina and Nanopore metagenomic reads (Unicycler) | **Short-read assembly (SPAdes)** | Short read contigs: 34,178 (9 samples) | Short read contigs: 16,930 (3 samples) |
|  | **Long read input** | 33,457 reads | 7,888 reads |
|  | **Assembly of long reads to short-read contigs (miniasm)** | Fully aligned reads: 5,401  Partially aligned reads: 25,960  Unaligned reads: 110,817  Total bases aligned: 99,888,567 bp  Mean alignment identity: 77.7%  Splits: 2,919 | Fully aligned reads: 392  Partially aligned reads: 1,782  Unaligned reads: 5,826  Total bases aligned: 9,293,553 bp  Mean alignment identity: 81.1%  Splits: 2,195 |
| Mapping (Bowtie2) | **Alignment rate range** | 6.46% - 40.17% | 18.55%-33.21% |
